# Dichlorido{*N*-[(5-methyl­thio­phen-2-yl)methyl­idene]-2-(pyridin-2-yl)ethanamine-κ^2^ *N*,*N*′}palladium(II)

**DOI:** 10.1107/S1600536812049240

**Published:** 2012-12-08

**Authors:** Mduduzi P. Radebe, Martin O. Onani, William M. Motswainyana

**Affiliations:** a Chemistry Department, University of the Western Cape, Private Bag X17, Bellville 7535, South Africa

## Abstract

In the title compound, [PdCl_2_(C_13_H_14_N_2_S)], the Pd^II^ ion is coordinated by two N atoms of the chelating bidentate ligand and two chloride anions, giving rise to a distorted square-planar geometry. The methyl-substituted thio­phene arm and the pyridine ring are connected to the metal cation through N atoms to form a six-membered chelate ring with a boat conformation, making the complex stable.

## Related literature

For the synthesis of imino-pyridyl ligands and their transition metal-based complexes, see: Onani & Motswainyana (2011 ▶); Motswainyana *et al.* (2011 ▶); Bianchini *et al.* (2010 ▶). For related structures, see: Motswainyana *et al.* (2012 ▶); Chen *et al.* (2007 ▶). For applications of these complexes, see: Ardizzoia *et al.* (2009 ▶); Tianpengfei *et al.* (2011 ▶).
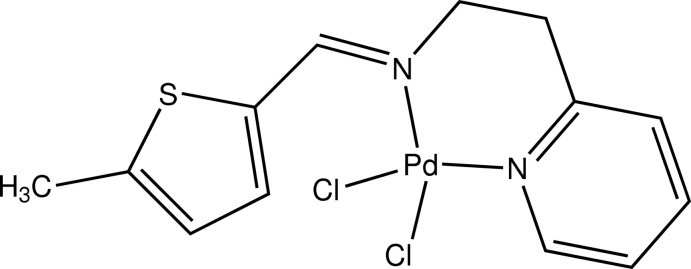


## Experimental

### 

#### Crystal data

[PdCl_2_(C_13_H_14_N_2_S)]
*M* *_r_* = 407.62
Monoclinic, 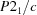

*a* = 12.0110 (5) Å
*b* = 9.1633 (4) Å
*c* = 13.6456 (6) Å
β = 97.930 (1)°
*V* = 1487.48 (11) Å^3^
*Z* = 4
Mo *K*α radiation
μ = 1.73 mm^−1^
*T* = 173 K
0.15 × 0.07 × 0.04 mm

#### Data collection

Bruker Kappa DUO APEXII diffractometer
Absorption correction: multi-scan (*SADABS*; Sheldrick, 1997 ▶) *T*_min_ = 0.781, *T*_max_ = 0.934
14701 measured reflections
3706 independent reflections
3129 reflections with *I* > 2σ(*I*)
*R*_int_ = 0.038

#### Refinement

*R*[*F* ^2^ > 2σ(*F* ^2^)] = 0.025
*wR*(*F* ^2^) = 0.056
*S* = 1.02
3706 reflections
173 parameters
H-atom parameters constrained
Δρ_max_ = 0.39 e Å^−3^
Δρ_min_ = −0.62 e Å^−3^

### 

Data collection: *APEX2* (Bruker, 2006 ▶); cell refinement: *SAINT* (Bruker, 2006 ▶); data reduction: *SAINT*; program(s) used to solve structure: *SHELXS97* (Sheldrick, 2008 ▶); program(s) used to refine structure: *SHELXL97* (Sheldrick, 2008 ▶); molecular graphics: *X-SEED* (Barbour, 2001 ▶); software used to prepare material for publication: *SHELXL97*.

## Supplementary Material

Crystal structure: contains datablock(s) I, global. DOI: 10.1107/S1600536812049240/bh2466sup1.cif

Structure factors: contains datablock(s) I. DOI: 10.1107/S1600536812049240/bh2466Isup2.hkl

Additional supplementary materials:  crystallographic information; 3D view; checkCIF report

## Figures and Tables

**Fig. 1.**
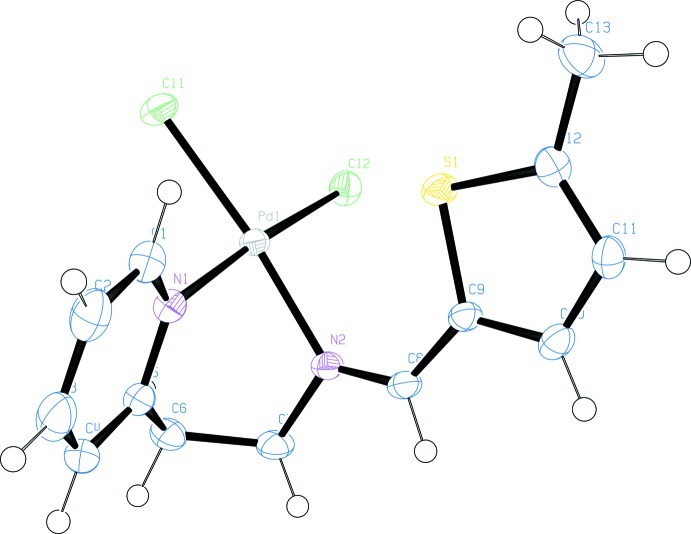
Molecular structure of the title complex showing 50% probability displacement ellipsoids for non-H atoms.

## supplementary crystallographic information

### Comment

The imino-pyridyl ligands coordinate as neutral, bidentate species when they are reacted with labile transition metal precursors to form air stable complexes, which show hemilability due to the weakly coordinating N atoms. The complexes could be investigated in various catalytic applications (Bianchini *et al.*, 2010; Motswainyana *et al.*, 2011; Ardizzoia *et al.*, 2009; Tianpengfei *et al.*, 2011).

In our study of imino-pyridyl Pd(II) complexes which could replace the expensive, air and moisture unstable phosphines for catalytic processes, we synthesized and crystallized the title compound (Fig. 1). The Pd atom is coordinated through the two N atoms of the ligand and two chloride anions, generating a distorted square planar coordination geometry around the Pd centre. The bond angles around the Pd metal atom of Cl2—Pd1—Cl1 [92.34 (2)°] and N2—Pd1—N1 [81.73 (7)°] show significant deviations from 90°, which confirms the distortion in the square planar geometry. The angles agree with those of similar compounds (Onani & Motswainyana, 2011; Motswainyana *et al.*, 2012). The Pd1—C11 bond lengths of 2.3016 (6) Å and Pd1—Cl2 of 2.3027 (6) Å are within the limits of the average Pd—Cl bond distance of 2.298 (15) Å for known palladium complexes (Chen *et al.*, 2007). The Pd—Cl bond distances are rather equal, indicating the absence of a *trans*-influence in the molecule.

### Experimental

To a suspension of PdCl_2_(cod) (0.0970 g, 0.34 mmol) in CH_2_Cl_2_ (5 ml) was added a solution of the ligand *N*-(5-methyl-thiophen-2-ylmethylene)-2-pyridineethanamine (0.0666 g, 0.34 mmol) in CH_2_Cl_2_ (10 ml). The yellow solution was stirred at room temperature for 8 h., resulting in the formation of a yellow precipitate. The precipitate was filtered, and recrystallization of the product from CH_2_Cl_2_ and an excess of C_6_H_14_ solution gave single crystals suitable for X-ray diffraction studies. The product yield was 76%.

### Refinement

All non-H atoms were refined anisotropically. All H atoms were placed in idealized positions and refined with constrained C—H distances of 0.95 (aromatic CH), 0.99 (methylene CH_2_) or 0.98 Å (methyl CH_3_). Isotropic displacement parameters for H atoms were calculated as *U*_iso_(H) = 1.2*U*_eq_(carrier C), except in the case of the methyl group, for which *U*_iso_(H) = 1.5*U*_eq_(C13).

### Crystal data

**Table:**

[PdCl_2_(C_13_H_14_N_2_S)]	*F*(000) = 808
*M**_r_* = 407.62	*D*_x_ = 1.820 Mg m^−^^3^
Monoclinic, *P*2_1_/*c*	Mo *K*α radiation, λ = 0.71073 Å
Hall symbol: -P 2ybc	Cell parameters from 14701 reflections
*a* = 12.0110 (5) Å	θ = 1.7–28.3°
*b* = 9.1633 (4) Å	µ = 1.73 mm^−^^1^
*c* = 13.6456 (6) Å	*T* = 173 K
β = 97.930 (1)°	Needle, yellow
*V* = 1487.48 (11) Å^3^	0.15 × 0.07 × 0.04 mm
*Z* = 4	

### Data collection

**Table:**

Bruker Kappa DUO APEXII diffractometer	3706 independent reflections
Radiation source: fine-focus sealed tube	3129 reflections with *I* > 2σ(*I*)
Graphite monochromator	*R*_int_ = 0.038
0.5° φ scans and ω scans	θ_max_ = 28.3°, θ_min_ = 1.7°
Absorption correction: multi-scan (*SADABS*; Sheldrick, 1997)	*h* = −16→16
*T*_min_ = 0.781, *T*_max_ = 0.934	*k* = −12→12
14701 measured reflections	*l* = −18→18

### Refinement

**Table:**

Refinement on *F*^2^	Primary atom site location: structure-invariant direct methods
Least-squares matrix: full	Secondary atom site location: difference Fourier map
*R*[*F*^2^ > 2σ(*F*^2^)] = 0.025	Hydrogen site location: inferred from neighbouring sites
*wR*(*F*^2^) = 0.056	H-atom parameters constrained
*S* = 1.02	*w* = 1/[σ^2^(*F*_o_^2^) + (0.0227*P*)^2^ + 0.6596*P*] where *P* = (*F*_o_^2^ + 2*F*_c_^2^)/3
3706 reflections	(Δ/σ)_max_ = 0.001
173 parameters	Δρ_max_ = 0.39 e Å^−^^3^
0 restraints	Δρ_min_ = −0.62 e Å^−^^3^
0 constraints	

### Fractional atomic coordinates and isotropic or equivalent isotropic displacement parameters (Å^2^)

**Table:**

	*x*	*y*	*z*	*U*_iso_*/*U*_eq_	
Pd1	0.253855 (14)	0.164855 (19)	0.101073 (12)	0.01838 (6)	
Cl1	0.35052 (5)	0.08399 (7)	0.24845 (4)	0.02846 (14)	
Cl2	0.08238 (5)	0.11869 (7)	0.15214 (4)	0.02567 (13)	
S1	0.18709 (5)	0.48733 (6)	0.13240 (4)	0.02374 (13)	
N1	0.39778 (16)	0.2026 (2)	0.04460 (14)	0.0229 (4)	
N2	0.18590 (15)	0.2349 (2)	−0.03402 (14)	0.0200 (4)	
C1	0.4789 (2)	0.2919 (3)	0.0894 (2)	0.0276 (5)	
H1	0.4720	0.3299	0.1531	0.033*	
C2	0.5710 (2)	0.3293 (3)	0.0452 (2)	0.0356 (6)	
H2	0.6260	0.3947	0.0768	0.043*	
C3	0.5820 (2)	0.2704 (4)	−0.0453 (2)	0.0397 (7)	
H3	0.6455	0.2940	−0.0768	0.048*	
C4	0.5005 (2)	0.1767 (3)	−0.0909 (2)	0.0346 (6)	
H4	0.5085	0.1342	−0.1530	0.042*	
C5	0.4071 (2)	0.1452 (3)	−0.04537 (18)	0.0248 (5)	
C6	0.3119 (2)	0.0495 (3)	−0.08966 (18)	0.0295 (6)	
H6A	0.3029	−0.0311	−0.0431	0.035*	
H6B	0.3316	0.0057	−0.1513	0.035*	
C7	0.1988 (2)	0.1301 (3)	−0.11346 (17)	0.0260 (5)	
H7A	0.1962	0.1824	−0.1772	0.031*	
H7B	0.1363	0.0588	−0.1196	0.031*	
C8	0.15436 (18)	0.3656 (3)	−0.05905 (17)	0.0208 (5)	
H8	0.1332	0.3829	−0.1277	0.025*	
C9	0.14775 (18)	0.4873 (3)	0.00534 (16)	0.0203 (5)	
C10	0.10937 (19)	0.6228 (3)	−0.02615 (18)	0.0240 (5)	
H10	0.0836	0.6448	−0.0935	0.029*	
C11	0.11169 (19)	0.7260 (3)	0.05032 (18)	0.0248 (5)	
H11	0.0878	0.8244	0.0401	0.030*	
C12	0.15202 (19)	0.6694 (3)	0.14103 (18)	0.0235 (5)	
C13	0.1685 (2)	0.7445 (3)	0.23892 (19)	0.0337 (6)	
H13A	0.1380	0.8436	0.2315	0.051*	
H13B	0.2490	0.7490	0.2638	0.051*	
H13C	0.1295	0.6900	0.2858	0.051*	

### Atomic displacement parameters (Å^2^)

**Table:**

	*U*^11^	*U*^22^	*U*^33^	*U*^12^	*U*^13^	*U*^23^
Pd1	0.02171 (9)	0.01803 (9)	0.01521 (9)	0.00063 (7)	0.00192 (6)	0.00031 (7)
Cl1	0.0322 (3)	0.0323 (3)	0.0194 (3)	0.0027 (2)	−0.0018 (2)	0.0039 (2)
Cl2	0.0246 (3)	0.0253 (3)	0.0277 (3)	0.0007 (2)	0.0059 (2)	0.0051 (2)
S1	0.0342 (3)	0.0184 (3)	0.0181 (3)	0.0019 (2)	0.0018 (2)	0.0024 (2)
N1	0.0241 (10)	0.0231 (10)	0.0212 (10)	0.0031 (8)	0.0020 (8)	0.0018 (8)
N2	0.0226 (9)	0.0210 (10)	0.0162 (9)	0.0002 (8)	0.0017 (7)	−0.0010 (8)
C1	0.0250 (12)	0.0270 (13)	0.0300 (14)	0.0025 (10)	0.0011 (10)	0.0013 (11)
C2	0.0246 (12)	0.0367 (16)	0.0445 (17)	0.0001 (11)	0.0011 (11)	0.0089 (13)
C3	0.0240 (13)	0.0556 (19)	0.0410 (17)	0.0075 (13)	0.0104 (12)	0.0203 (15)
C4	0.0353 (14)	0.0474 (17)	0.0226 (13)	0.0178 (13)	0.0094 (11)	0.0080 (12)
C5	0.0291 (12)	0.0252 (12)	0.0203 (12)	0.0087 (10)	0.0036 (9)	0.0031 (10)
C6	0.0392 (14)	0.0271 (13)	0.0220 (13)	0.0062 (11)	0.0034 (10)	−0.0071 (10)
C7	0.0345 (13)	0.0254 (13)	0.0171 (12)	−0.0009 (10)	−0.0005 (10)	−0.0060 (9)
C8	0.0203 (10)	0.0273 (13)	0.0150 (11)	−0.0006 (9)	0.0025 (8)	0.0022 (9)
C9	0.0204 (11)	0.0247 (12)	0.0163 (11)	0.0007 (9)	0.0039 (8)	0.0038 (9)
C10	0.0231 (11)	0.0264 (12)	0.0226 (12)	0.0010 (9)	0.0033 (9)	0.0077 (10)
C11	0.0232 (11)	0.0204 (12)	0.0318 (14)	0.0027 (9)	0.0073 (10)	0.0045 (10)
C12	0.0251 (11)	0.0186 (11)	0.0281 (13)	−0.0014 (9)	0.0083 (9)	0.0018 (10)
C13	0.0490 (16)	0.0222 (13)	0.0319 (15)	−0.0002 (12)	0.0123 (12)	−0.0023 (11)

### Geometric parameters (Å, º)

**Table:**

Pd1—N2	2.0152 (18)	C5—C6	1.500 (3)
Pd1—N1	2.017 (2)	C6—C7	1.541 (3)
Pd1—Cl1	2.3016 (6)	C6—H6A	0.9900
Pd1—Cl2	2.3027 (6)	C6—H6B	0.9900
S1—C12	1.729 (2)	C7—H7A	0.9900
S1—C9	1.733 (2)	C7—H7B	0.9900
N1—C1	1.352 (3)	C8—C9	1.429 (3)
N1—C5	1.355 (3)	C8—H8	0.9500
N2—C8	1.288 (3)	C9—C10	1.373 (3)
N2—C7	1.472 (3)	C10—C11	1.406 (3)
C1—C2	1.374 (4)	C10—H10	0.9500
C1—H1	0.9500	C11—C12	1.368 (3)
C2—C3	1.371 (4)	C11—H11	0.9500
C2—H2	0.9500	C12—C13	1.491 (3)
C3—C4	1.384 (4)	C13—H13A	0.9800
C3—H3	0.9500	C13—H13B	0.9800
C4—C5	1.385 (4)	C13—H13C	0.9800
C4—H4	0.9500		
			
N2—Pd1—N1	81.73 (7)	C5—C6—H6B	108.8
N2—Pd1—Cl1	173.54 (6)	C7—C6—H6B	108.8
N1—Pd1—Cl1	91.91 (6)	H6A—C6—H6B	107.7
N2—Pd1—Cl2	93.96 (5)	N2—C7—C6	109.68 (19)
N1—Pd1—Cl2	175.20 (6)	N2—C7—H7A	109.7
Cl1—Pd1—Cl2	92.34 (2)	C6—C7—H7A	109.7
C12—S1—C9	91.90 (12)	N2—C7—H7B	109.7
C1—N1—C5	120.0 (2)	C6—C7—H7B	109.7
C1—N1—Pd1	122.21 (17)	H7A—C7—H7B	108.2
C5—N1—Pd1	117.53 (16)	N2—C8—C9	127.0 (2)
C8—N2—C7	117.9 (2)	N2—C8—H8	116.5
C8—N2—Pd1	127.30 (16)	C9—C8—H8	116.5
C7—N2—Pd1	113.28 (15)	C10—C9—C8	123.9 (2)
N1—C1—C2	121.7 (3)	C10—C9—S1	110.26 (18)
N1—C1—H1	119.1	C8—C9—S1	125.80 (17)
C2—C1—H1	119.1	C9—C10—C11	113.9 (2)
C3—C2—C1	118.7 (3)	C9—C10—H10	123.1
C3—C2—H2	120.6	C11—C10—H10	123.1
C1—C2—H2	120.6	C12—C11—C10	112.6 (2)
C2—C3—C4	119.9 (3)	C12—C11—H11	123.7
C2—C3—H3	120.0	C10—C11—H11	123.7
C4—C3—H3	120.0	C11—C12—C13	128.5 (2)
C3—C4—C5	119.6 (3)	C11—C12—S1	111.32 (18)
C3—C4—H4	120.2	C13—C12—S1	120.16 (18)
C5—C4—H4	120.2	C12—C13—H13A	109.5
N1—C5—C4	119.9 (2)	C12—C13—H13B	109.5
N1—C5—C6	116.0 (2)	H13A—C13—H13B	109.5
C4—C5—C6	124.1 (2)	C12—C13—H13C	109.5
C5—C6—C7	114.0 (2)	H13A—C13—H13C	109.5
C5—C6—H6A	108.8	H13B—C13—H13C	109.5
C7—C6—H6A	108.8		
			
N2—Pd1—N1—C1	124.39 (19)	N1—C5—C6—C7	64.5 (3)
Cl1—Pd1—N1—C1	−56.80 (18)	C4—C5—C6—C7	−115.2 (3)
N2—Pd1—N1—C5	−49.93 (17)	C8—N2—C7—C6	132.5 (2)
Cl1—Pd1—N1—C5	128.88 (16)	Pd1—N2—C7—C6	−34.5 (2)
N1—Pd1—N2—C8	−94.6 (2)	C5—C6—C7—N2	−39.7 (3)
Cl2—Pd1—N2—C8	87.59 (19)	C7—N2—C8—C9	−173.1 (2)
N1—Pd1—N2—C7	71.03 (16)	Pd1—N2—C8—C9	−8.1 (3)
Cl2—Pd1—N2—C7	−106.83 (15)	N2—C8—C9—C10	−177.5 (2)
C5—N1—C1—C2	1.3 (4)	N2—C8—C9—S1	3.0 (4)
Pd1—N1—C1—C2	−172.90 (19)	C12—S1—C9—C10	−0.28 (18)
N1—C1—C2—C3	−2.0 (4)	C12—S1—C9—C8	179.2 (2)
C1—C2—C3—C4	0.7 (4)	C8—C9—C10—C11	−179.3 (2)
C2—C3—C4—C5	1.2 (4)	S1—C9—C10—C11	0.2 (3)
C1—N1—C5—C4	0.7 (3)	C9—C10—C11—C12	0.0 (3)
Pd1—N1—C5—C4	175.19 (18)	C10—C11—C12—C13	179.1 (2)
C1—N1—C5—C6	−179.0 (2)	C10—C11—C12—S1	−0.2 (3)
Pd1—N1—C5—C6	−4.5 (3)	C9—S1—C12—C11	0.27 (19)
C3—C4—C5—N1	−2.0 (4)	C9—S1—C12—C13	−179.1 (2)
C3—C4—C5—C6	177.7 (2)		
